# Protective Effects of Alpha Lipoic Acid Against Ionizing Radiation-Induced Hepatotoxicity in Rats

**DOI:** 10.5152/eurasianjmed.2023.0148

**Published:** 2023-06-01

**Authors:** Arzu Gezer, Abubekir Laloglu, Meltem Kirli Bölükbaş

**Affiliations:** 1 Atatürk University, Vocational School of Health Services, Erzurum, Turkey; 2 Ataturk University, Faculty of Dentistry, Oral, Dental and Maxillofacial Radiology, Erzurum, Turkey; 3 Department of Radiation Oncology, Health Sciences University Erzurum Regional Training and Research Hospital, Yakutiye-Erzurum, Turkey

**Keywords:** Alpha lipoic acid, liver, radiation, rat

## Abstract

**Objective:**

Radiation is used to treat cancer but causes serious complications, such as liver toxicity. In this study, the protective effects of alpha lipoic acid against the unwanted effects of radiation used in many cancer treatments which can cause damage after treatment were investigated.

**Material and Methods:**

The sample consisted of 32 Sprague–Dawley male rats randomized equally into 4 groups. The control group received no intervention. The alpha lipoic acid group was administered 50 mg/kg (dissolved in 0.9% NaCl) for 3 days. The ionizing radiation group was exposed to a total of 30 Gy radiation in 10 Gy fractions per day. The ionizing radiation + alpha lipoic acid group was administered 50 mg/kg alpha lipoic acid® prior to exposure to a total of 30 Gy radiation in 10 Gy fractions per day. Rats were sacrificed by cervical dislocation, and the liver was removed for histopathological studies and superoxide dismutase and malondialdehyde assays. Liver tissues were histopathologically assessed using hematoxylin-eosin staining after 4 weeks of the experiment.

**Results:**

The ionizing radiation + alpha lipoic acid group had significantly less severe necrosis than the ionizing radiation group. Compared to the ionizing radiation group and the ionizing radiation + alpha lipoic acid group, superoxide dismutase enzyme activity was decreased with the addition of alpha lipoic acid. In addition, when the amount of malondialdehyde, which is a marker of oxidative stress, was examined, it was determined that the amount of malondialdehyde in the ionizing radiation + alpha lipoic acid group was lower than in the ionizing radiation Group.

**Conclusion:**

Alpha lipoic acid® mitigates radiotherapy-induced damage in liver tissue.

**Main Points**

- Ionizing radiation therapy can cause damage to healthy liver tissue.
- Alpha lipoic acid can be used to reduce radiation-induced liver damage.
- We hope that if our study is supported by experimental studies in the future, alpha lipoic acid will find a new indication area.

## Introduction

Radiotherapy (RT) is a type of cancer treatment involving high doses of x-rays or other types of energy to kill cancer cells. Radiation is also referred to as ionizing radiation (IR) because it passes through tissues and ionizes atoms and molecules by removing electrons from them. Ionizing radiation can change genes or kill cells, which stops cell growth. The extent of radiation-induced damage depends on the dose of the radiation. It is possible to increase success rate in tumor control with increasing doses. However, the higher radiation dose means higher risk of complications in healthy tissues. Ionizing radiation affects healthy tissues directly or indirectly.^1^ It not only affects the target molecule, DNA (direct effect), but also ionizes cellular atoms and causes molecular degradation (indirect effect).^2^ Ionizing radiation affects tissues and cells in different ways.^3^ It causes an increase in the number of free oxygen radicals, which adversely affect cellular membrane lipids, proteins, DNA, and the antioxidant defense mechanisms.^4^ Therefore, radiation toxicity is a common side effect associated with reactive oxygen radicals causing oxidative damage.^5^ Antioxidants suppressing free oxygen radicals can reduce radiation-induced tissue damage. There is a growing body of research on radiation radioprotectors to minimize radiation-induced oxidative damage.^6^ The higher the radiation dose, the greater the cell cycle disruption leading to abnormal mitosis or cell death.^7^

The liver is the largest and an important organ in the human body. It is responsible for producing bile, storing glycogen, protein synthesis, production of hormones and enzymes necessary for digestion, ensuring the absorption of food metabolites, eliminating waste products, and breaking down red blood cells. Due to its physiological and biochemical functions, it is exposed to numerous toxicities and medications.^8^ The liver has a low tolerance to radiation. However, a part of the liver may be exposed to a certain dose of radiation in the distal esophagus, upper abdomen, right lung, and whole abdomen or whole body.^9,10^ Irradiation causes liver diseases, such as hepatitis and hepatocyte death.^11^

Laboratory experiments on animals allow us to work with populations with minimal individual variability and test appropriate doses of radiation and active substances and evaluate their biological effects. Moreover, laboratory experiments on animals provide insight into the effects and underlying mechanisms of IR because in vivo systems may react differently to radiation than in vitro systems.^12^

Many people undergo radiotherapy for cancer every year. Therefore, the effect of radiation on organs has been a matter of curiosity. Researchers have sought ways to prevent both acute and late toxicity due to radiation-induced toxicity that can sometimes be much worse than the first lesion for which treatment was received. Therefore, this study histopathologically and biochemically investigated the protective effects of alpha lipoic acid® (α-LA) against IR-induced damage to liver tissues in rats.

## Materials and Methods

The study was approved by the Local Ethics Committee on Animal Experiments of our university (No: 8/215). The experiment adhered to the criteria outlined by the European Community Guidelines. The sample consisted of 32 Sprague–Dawley male rats (250 ± 20 g) bred at the Medical Experimental Application and Research Center of our university. The rats were kept in plastic cages under standard laboratory conditions at a constant temperature of 19-21˚C for 12 hours of light/darkness throughout the experiment. They were fed with pellet feed *ad libitum* throughout the experiment. 

Alpha lipoic acid® was supplied from Solgar Inc. Co. Ilko (Pharmaceutical Industry and Trade Inc., Turkey). It was dissolved in 0.9% NaCl and mixed until homogenized to produce α-LA suspension. Alpha lipoic acid® was administered by gavage to rats (50 mg/kg) in the α-LA group once daily for 3 days. Rats in the IR+ α-LA group were administered α-LA by gavage (50 mg/kg) for 3 days prior to exposure to radiotherapy.^13^

Rats were randomly classified into 4 experimental groups (groups 1-4): Control group received no therapy and only food and water; α-LA group was administered with 50 mg/kg α-LA ®, IR group was administered with ionized radiation (30 Gy), and IR+ α-LA group received both IR and α-LA, respectively (Table 1). The IR and IR+ α-LA groups were irradiated using a linear accelerator (Elekta Synergy®) at the Department of Radiation Oncology. Total body irradiation was performed using x-rays in a box made of wood with a size of 30 × 30 × 5 cm and a depth of 3 cm. The rats were mildly sedated with Sevoflurane (Sevorane®, Abbott Lab. Istanbul, Turkey) and irradiated in groups. A perforated tray was placed at the top of the box to allow the rats to get oxygen during irradiation. The total body irradiation was performed for 3 days with daily 10 Gy/fraction irradiation.

### Histological Analyses

The rats were administered 20 mg/kg thiopental sodium and 5% sevoflurane inhalation anesthesia on the 30th day of the experiment. Necropsy was performed to collect liver tissues, which were fixed in 10% neutral formalin solution. Tissues were dehydrated in ascending grades of alcohol (50%, 70%, 90%, and 100%) for 3 hours and cleared in 2 changes of xylene (clearing agent) for 3 hours. The tissues were then infiltrated and embedded in paraffin wax (Sigma Aldrich), cut with a rotary microtome (Leica, RM2255), and stained using hematoxylin and eosin (H&E) staining technique.^14^ Hepatocytes in 6 random regions were semi-quantitative as necrotic and degenerative; it was evaluated under light microscope as no (−), light (+), moderate (++), and (+++) by using the image analysis computer program named Image J 1.43, according to the intensity of staining: none (0), mild (1), moderate (2), and severe (3).^15^

### Biochemical Analyses

The prescribed method for the determination of superoxide dismutase (SOD) activity proposed by Mc Cord and Fridovich^16^ was followed Superoxide dismutase enzyme activity was expressed as U/mg protein. Malondialdehyde (MDA) analysis was determined using the method proposed by Draper and Hadley^17^ and the results were given as nmol/g.

All data were analyzed using the Statistical Package for Social Sciences v. 20 (SPSS) (IBM. Corp., Armonk, NY, USA) at a significance level of *P* < .05. The Kruskal–Wallis test was used to determine between-group differences. The Mann–Whitney *U*-test was used for comparisons.

## Results

**Figure 1.**
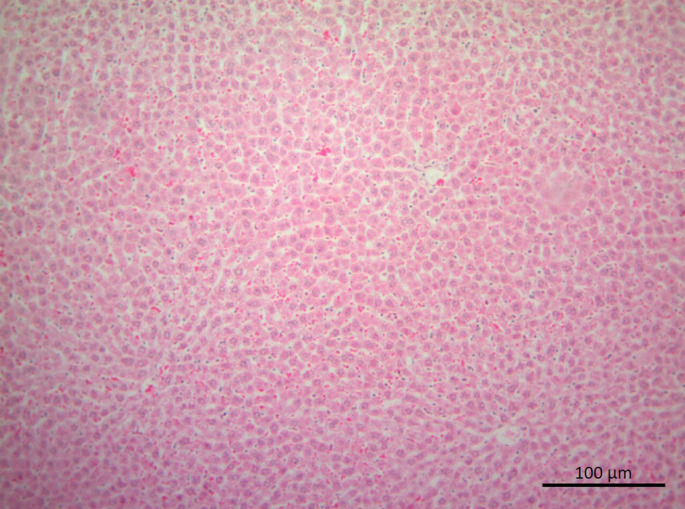
Control group, normal histopathology. ×100-H&E. H&E, hematoxylin and eosin.

**Figure 2.**
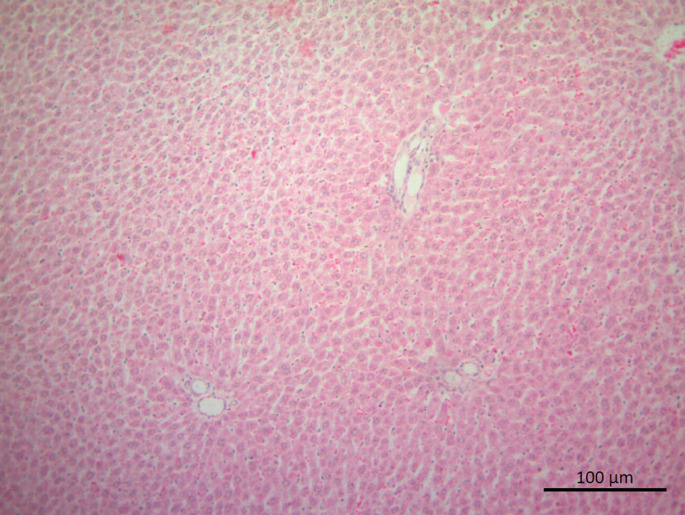
α-LA group, normal histopathology. ×100-H&E. α-LA, alpha lipoic acid; H&E, hematoxylin and eosin.

**Figure 3.**
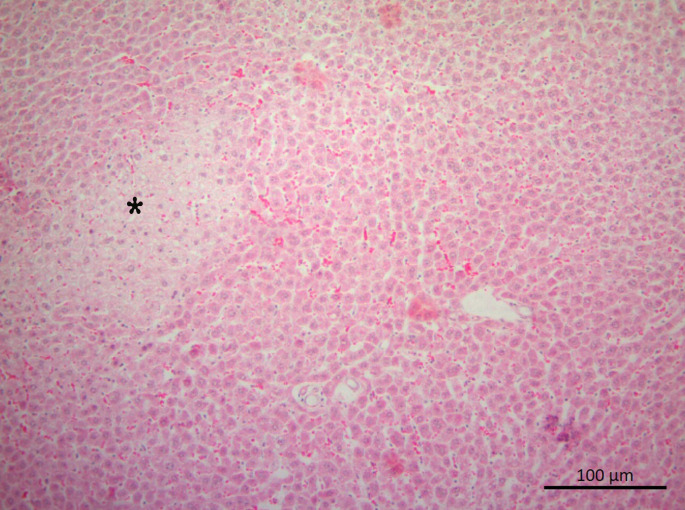
IR group, severe necrosis in hepatocytes (*).×100-H&E. IR, ionizing radiation; H&E, hematoxylin and eosin.

**Figure 4.**
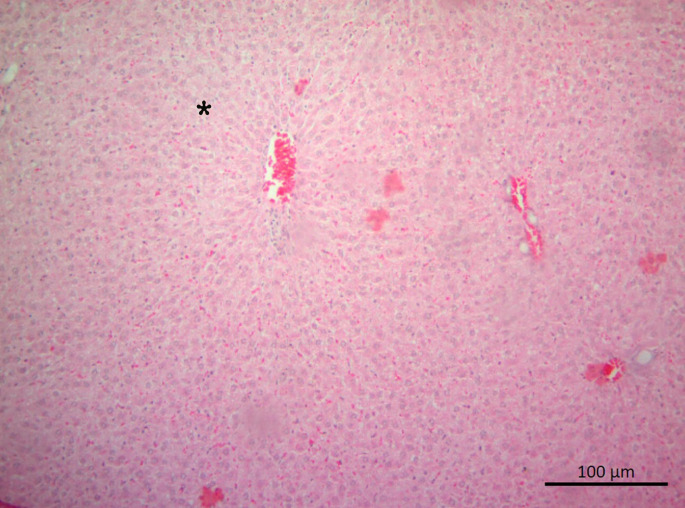
IR+ α-LA group, mild necrosis in hepatocytes (*).×100-H&E. H&E, hematoxylin and eosin; α-LA, alpha lipoic acid; IR, ionizing radiation.

Alpha lipoic acid® did not adversely affect the liver parenchyma. Control animals and α-LA group exhibit similar histological properties, which show normal histology with virtually intact central vein and healthy-looking hepatocytes (*P *> .05) (Figure 1 and 2). In the IR group, extensive necrosis was observed in hepatocytes (Figure 3). The IR+ α-LA group had significantly less severe necrosis than the IR group as shown in Figure 4. However, there were significant differences between the groups (*P *< .05) as presented in Table 2.

**Figure 5.**
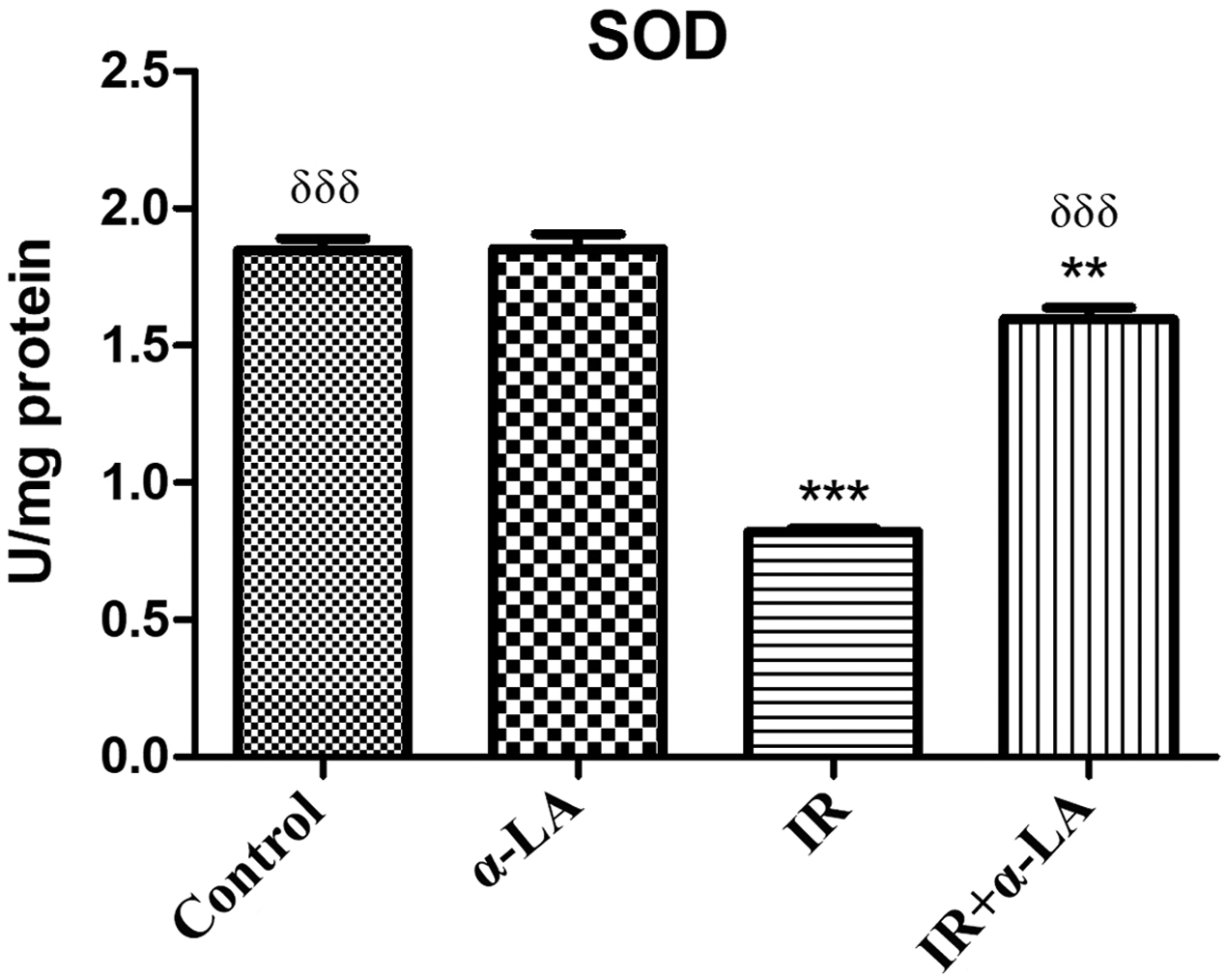
SOD values of groups. α-LA, alpha lipoic acid; IR, ionized radiation; IR+ α-LA, ionized radiation + alpha lipoic acid; SOD, superoxide dismutase. *Analysis of SOD values compared to the control group. ^δ^Analysis of SOD values compared to the IR group. Significant difference, *P* < .05.

When we examined the SOD values of our study, it was seen that the SOD values in the IR group were significantly lower than the control group (*P* < .001). In the IR+ α-LA group, on the other hand, it was observed that the decreased SOD values due to IR increased significantly with the administration of α-LA (*P* < .001) (Figure 5). Superoxide dismutase enzyme values of rats are presented in Table 3. 

**Figure 6.**
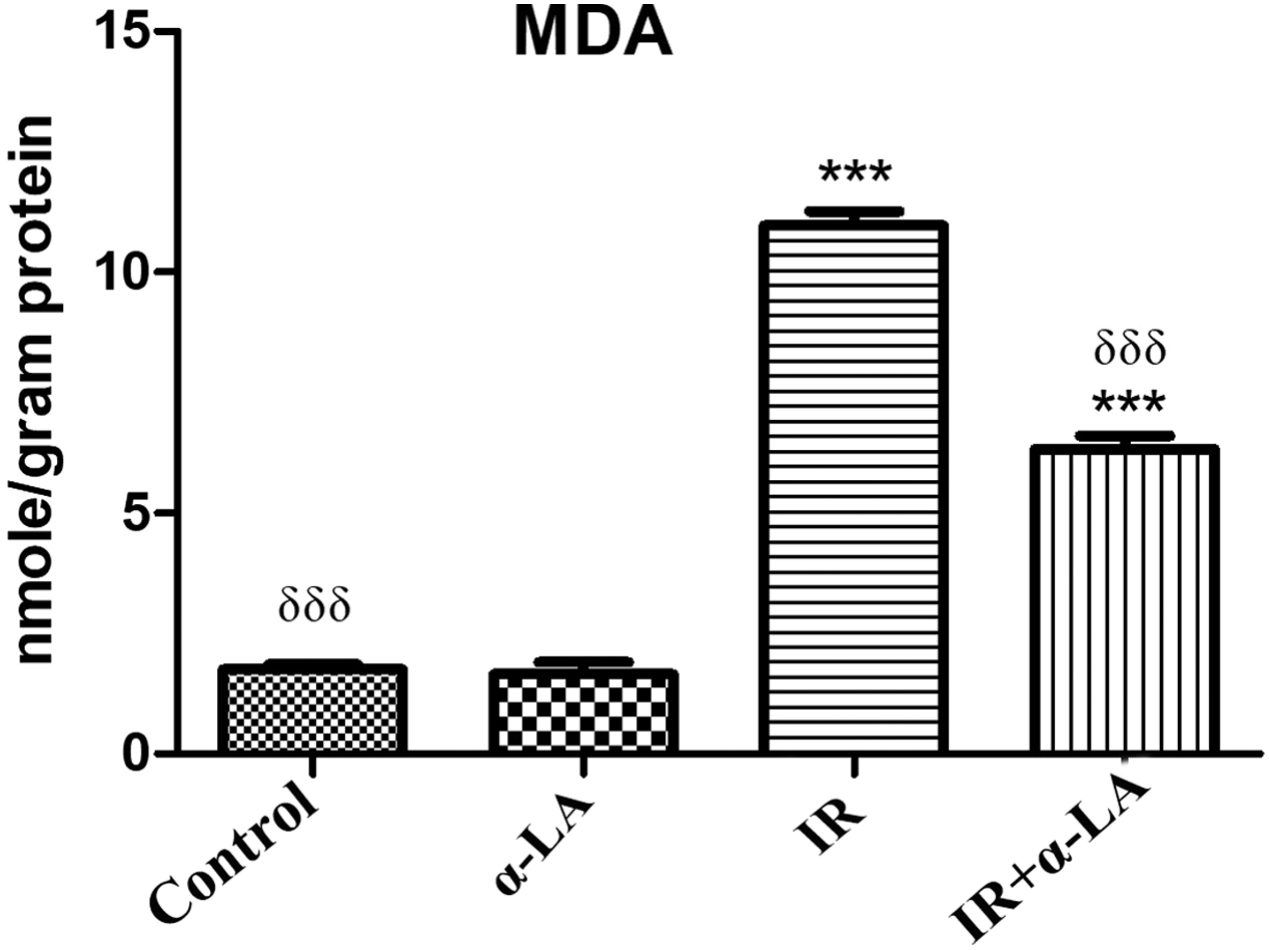
MDA values of groups. α-LA, alpha lipoic acid; IR, ionized radiation; IR+ α-LA, ionized radiation + alpha lipoic acid; MDA, malondialdehyde. *Analysis of MDA values compared to the control group. ^δ^Analysis of MDA values compared to the IR group. Significant difference, *P* < .05).

When we examined the MDA values of our study, it was seen that the MDA values in the IR group increased significantly compared to the control group (*P* < .001). In the IR+ α-LA group, it was observed that the increased MDA values due to IR decreased significantly with α-LA administration (*P* < .001) (Figure 6). Malondialdehyde values of rats are presented in Table 3.

## Discussion

Radiation causes protein oxidation, lipid peroxidation, DNA chain breaks, as well as macromolecular changes. It also attacks certain cellular components and promotes various conditions, such as genomic instability and DNA damage, resulting in tissue damage.^18^ Despite all the advances in radiotherapy, the early and late side effects of RT should never be ignored due to the effects of RT on normal healthy tissues. In some cases, the dose of RT cannot be given as high as desired because normal healthy tissue is very radiosensitive.^19^

Histological examination of the liver tissue of rat was carried out in this study which supports the biochemical findings after administering a 30 Gy dose of total body radiation. There was observed cellular damage and necrosis in hepatocytes in liver tissues. Karahan et al^20^ reported an increase in the number of binucleated hepatocytes and a reduction in the number of proliferating hepatocytes in the liver of irradiated rats (10 Gy). Kim and Yung^21^ determined that radiation caused fibrotic changes and cell damage in rodent livers. Cheema et al^22^ observed metabolic changes in the liver tissues and slight changes in the other tissues of primates exposed to total body radiation (7.2 Gy). The findings of the present study indicated that the IR and α-LA group rat shows mild necrosis in hepatocytes which is an indication of pathologic improvement in most accumulative doses.

Hepatocytes are parenchymal cells that play an important role in many of their metabolic functions and account for approximately 80% of the formations in the liver. Radiation-induced toxicity causes dysfunctions and different lesions in hepatic cells.^23^ The liver is a vital organ that is responsible for disposing of toxic metabolites and medications. The liver is mostly included in the RT field in the irradiation of upper abdomen malignancies. For this reason, some or all of the liver is exposed to the RT dose at certain rates.^24^

In our study, it was observed that SOD activity was higher in the IR group than in the IR+ α-LA group which shows that histological damage induced in the liver of irradiated rats was associated with the decrease in the activity of the antioxidant enzymes SOD. It was also observed that the amount of MDA was higher in the IR group than in the IR+ α-LA group. High levels of MDA, which is a determinant of lipid peroxidation, indicate that it causes cell damage. The results of our study are in accordance with the findings in a study by Saada et al^25^, which suggested that histological damage induced in the liver of irradiated rats was associated with an increase in the content of lipid peroxides and a decrease in the activity of the antioxidant enzymes SOD and catalase. In addition, exposure to radiation causes injury to blood vessels provoking anoxia of tissues with degeneration and necrosis of hepatic parenchyma.^26^ Also, cytoplasmic changes such as swelling, vacuolization, and alteration in the various components of the plasma membrane were seen.^27^ Based on this, radiation is said to induce liver cell damage and is significantly inhibited by α-LA. The result of the present study shows that α-LA has protective properties against the undesirable effects of radiation due to the presence of natural antioxidant in α-LA.

Many cancer patients undergoing radiotherapy suffer from liver tissue damage because it enters the radiation field. The effects of IR on the organism depend on many factors that should be addressed from a broad spectrum. Limitations of this research include low access to funds and other high throughput equipment and molecular techniques to study the possible mutations induced by radiation. In conclusion, we can state that α-LA can be used to reduce radiation-induced liver damage. However, further preclinical and clinical research is warranted to better understand its molecular basis and effects and find answers to its toxicity, tolerability, and dose.

## Figures and Tables

**Table 1.** Experimental Groups

Groups	Alpha Lipoic Acid (mg/kg)	Radiation (Gy)
Control	No	No
α-LA	50	No
IR	No	30
IR+α-LA	50	30

α-LA, alpha lipoic acid (50 mg/kg); IR, ionized radiation (30 Gy); IR+α-LA, ionized radiation (30 Gy) + alpha lipoic acid (50 mg/kg).

**Table 2.** Histopathological Evaluation of Liver Tissue

Groups	Necrosis in Hepatocytes
Control	0.33 ± 0.51^a^
α-LA	0.33 ± 0.51^a^
IR	2.83 ± 0.40^b^
IR+α-LA	1.83 ± 0.40^c^

α-LA, alpha lipoic acid; IR, ionized radiation; IR+α-LA, ionized radiation + alpha lipoic acid.^ a,b,c^Between-group differences (*P < .*05).

**Table 3.** SOD Enzyme Values and MDA Values in Rats of Given Groups (U/L)

Groups	SOD	MDA
Control	1.9	1.8
α-LA	1.99	1.2
IR	0.82	10.39
IR+α-LA	1.61	6.07

α-LA, alpha lipoic acid; IR, ionized radiation; IR+α-LA, ionized radiation + alpha lipoic acid; SOD, superoxide dismutase; MDA, malondialdehyde.

^a,b,c^Between-group differences (*P *< .05).
